# Auranofin Induces Lethality Driven by Reactive Oxygen Species in High-Grade Serous Ovarian Cancer Cells

**DOI:** 10.3390/cancers15215136

**Published:** 2023-10-25

**Authors:** Farah H. Abdalbari, Elvis Martinez-Jaramillo, Benjamin N. Forgie, Estelle Tran, Edith Zorychta, Alicia A. Goyeneche, Siham Sabri, Carlos M. Telleria

**Affiliations:** 1 Experimental Pathology Unit, Department of Pathology, Faculty of Medicine and Health Sciences, McGill University, Montreal, QC H3A 2B4, Canada; farah.abdalbari@mail.mcgill.ca (F.H.A.); elvis.martinez-jaramillo@mail.mcgill.ca (E.M.-J.); benjamin.forgie@mail.mcgill.ca (B.N.F.); estelletran@rcsi.ie (E.T.); edith.zorytcha@mcgill.ca (E.Z.); alicia.goyeneche@affiliate.mcgill.ca (A.A.G.); 2 Cancer Research Program, Research Institute, McGill University Health Centre, Montreal, QC H4A 3J1, Canada; siham.sabri.ab@outlook.com

**Keywords:** auranofin, high-grade serous ovarian cancer, TrxR, apoptosis, DNA damage, ROS, L-buthionine sulfoximine, cisplatin, N-acetyl cysteine, drug repurposing, GSH

## Abstract

**Simple Summary:**

High-grade serous ovarian cancer (HGSOC) is the most prevalent type of ovarian cancer, accounting for 70% of ovarian cancer deaths. This is primarily due to the development of resistance against standard platinum-based chemotherapy. Several drugs are currently undergoing repurposing against ovarian cancer, including auranofin (AF), an anti-rheumatoid agent. The mechanism of action of AF has been studied in various cancers, however, there have been fewer studies on the effects of AF in HGSOC. In this study, we explore the mechanisms of action of AF in human HGSOC cells that are sensitive or resistant to platinum. We demonstrate the various cytotoxic effects of AF in HGSOC via the targeting of multiple pathways, suggesting the potential use of AF in a long-term consolidation therapy against this disease.

**Abstract:**

High-grade serous ovarian cancer (HGSOC) accounts for 70% of ovarian cancer cases, and the survival rate remains remarkably low due to the lack of effective long-term consolidation therapies. Clinical remission can be temporarily induced by platinum-based chemotherapy, but death subsequently results from the extensive growth of a platinum-resistant component of the tumor. This work explores a novel treatment against HGSOC using the gold complex auranofin (AF). AF primarily functions as a pro-oxidant by inhibiting thioredoxin reductase (TrxR), an antioxidant enzyme overexpressed in ovarian cancer. We investigated the effect of AF on TrxR activity and the various mechanisms of cytotoxicity using HGSOC cells that are clinically sensitive or resistant to platinum. In addition, we studied the interaction between AF and another pro-oxidant, L-buthionine sulfoximine (L-BSO), an anti-glutathione (GSH) compound. We demonstrated that AF potently inhibited TrxR activity and reduced the vitality and viability of HGSOC cells regardless of their sensitivities to platinum. We showed that AF induces the accumulation of reactive oxygen species (ROS), triggers the depolarization of the mitochondrial membrane, and kills HGSOC cells by inducing apoptosis. Notably, AF-induced cell death was abrogated by the ROS-scavenger N-acetyl cysteine (NAC). In addition, the lethality of AF was associated with the activation of caspases-3/7 and the generation of DNA damage, effects that were also prevented by the presence of NAC. Finally, when AF and L-BSO were combined, we observed synergistic lethality against HGSOC cells, which was mediated by a further increase in ROS and a decrease in the levels of the antioxidant GSH. In summary, our results support the concept that AF can be used alone or in combination with L-BSO to kill HGSOC cells regardless of their sensitivity to platinum, suggesting that the depletion of antioxidants is an efficient strategy to mitigate the course of this disease.

## 1. Introduction

Ovarian cancer is the eighth leading cause of cancer-related deaths among women worldwide [1]. According to GLOBOCAN, there were 313,959 new ovarian cancer cases and 207,252 deaths due to ovarian cancer in 2020 [2]. Over the past few decades, there has been a small reduction in the incidence of ovarian cancer, primarily due to preventative measures, such as the introduction of oral contraceptives [3], and the decline in the use of menopausal hormonal therapy [4]. In contrast, there has been minimal improvement in the overall survival of patients with this disease [1,5]. Treatment with platinating agents is very efficient at the beginning of the illness following diagnosis and debulking surgery, leading to an initial response rate of 80% [6,7]. However, over time the disease almost always recurs with a platinum resistant phenotype that is extremely challenging to treat, thus explaining its high mortality [8]. Acquired platinum resistance occurs via multipronged mechanisms with decreased intracellular accumulation, increased drug detoxification, and the increased activity of the DNA repair machinery among the most often investigated [5]. 

To reduce the high mortality of ovarian cancer, platinum-based chemotherapy needs to be coupled with an additional treatment that can be given chronically to maintain the disease in a dormant stage. Drugs with cytotoxic activity that are currently approved for treating other conditions are ideal candidates for this role. Drug repurposing is a cost-effective approach in which drugs approved for one condition can be administered for a different disorder [9,10]. With this goal in mind, our laboratory has shown that the antiprogesterone/antiglucocorticoid agent mifepristone is efficient as an upfront therapy or after cisplatin and/or paclitaxel therapy against ovarian cancer cells [5,11,12,13,14]. We have also shown the efficacy of the HIV inhibitor nelfinavir against ovarian cancer cells sensitive or resistant to platinum [15]. Here, we added auranofin, a gold complex approved in 1985 to treat rheumatoid arthritis [16], to the list of anti-ovarian cancer drugs emerging from repurposed agents. 

There have been studies exploring the cytotoxic effect of auranofin in ovarian cancer. However, such studies were conducted using cell lines that do not represent the high-grade serous histotype we report here. For instance, studies have used A2780 cells [17,18], which have been identified as representing an endometrial ovarian carcinoma [19]; OV2008 [20], which have been reported to be misidentified [21] and likely of a cervical nature [22]; SKOV-3 [23,24], which have been genetically identified to represent a clear ovarian adenocarcinoma [19]; and OVCAR-5 [24], which were incorrectly identified as being of ovarian origin while they are actually of a gastrointestinal nature [25]. Finally, other studies have used the platinum-resistant OV2008 (C13*) [20,26], which are cells that were developed in vitro from OV2008 cells after a dose-escalating exposure to cisplatin. In this work we explore the cytotoxicity of auranofin towards a pair of cell lines that are, respectively, sensitive or resistant to platinum after being isolated from a patient when she was clinically sensitive or resistant to the drug [27]; these cells have been demonstrated to genomically represent the most prevalent histotype of the disease: high-grade serous ovarian cancer (HGSOC) [8,28]. 

The primary mechanism of action of auranofin is to act as a pro-oxidative agent, increasing the production of reactive oxygen species (ROS) as a consequence of inhibiting the thioredoxin reductase (TrxR) anti-oxidant system [29]. TrxR is overexpressed in various cancers, including non-small cell lung cancer [30], breast cancer [31], and cisplatin-resistant ovarian cancer [20]. The TrxR system is involved in the overall promotion of tumor progression by preventing cell death triggered by oxidative stress [32]. Of interest, TrxR overexpression is associated with a shorter overall survival in patients with ovarian cancer based on a Kaplan–Meier survival analysis [32]. These findings suggest that TrxR is an attractive therapeutic target against ovarian cancer, and auranofin is a potent TrxR inhibitor and pro-oxidative agent that can be used to combat this disease. Previous reports on various cancer cells have demonstrated that auranofin induces the inhibition of cell proliferation by causing the overproduction of ROS [33], caspase-independent apoptosis [34], and cell death triggered by DNA damage [35]. Additionally, auranofin has been shown to inhibit angiogenesis [36], protein homeostasis [37,38], and deubiquitinases involved in proteasome-mediated protein degradation [39]. These findings indicate that auranofin is a potent anti-cancer agent that negatively targets the multiple metabolic pathways of cancer cells. In this study, we identified the mechanisms of cytotoxicity induced by auranofin in HGSOC cells that have different clinical sensitivities to platinum. We show that auranofin causes the ROS-dependent inhibition of cell proliferation, caspase-associated apoptosis, mitochondrial membrane depolarization, DNA damage, the cleavage of poly-ADP ribose polymerase (PARP), and the polyubiquitination of proteins [39]. Additionally, we show a synergistic lethal interaction between auranofin and a second pro-oxidant agent, the glutathione (GSH) inhibitor, L-buthionine sulfoximine (L-BSO); this drug interaction, involving two blockers of key antioxidant pathways that cancer cells rely upon, is dependent on the presence of ROS. 

## 2. Materials and Methods

### 2.1. Reagents and Cell Lines

PEO1 cells are epithelial ovarian cancer cells isolated from a patient after their first relapse 22 months following treatment with cisplatin, 5-fluorouracil, and chlorambucil, while the patient was still sensitive to platinum-based chemotherapy. PEO4 cells were subsequently isolated from the same patient after the second relapse in which the patient was no longer sensitive to chemotherapy. These cell lines were histologically characterized in 1988 and sequenced in 2010 [27,28], whereas we authenticated them in 2020 based on their autosomal short-tandem repeats [15]. The cells were cultured in RPMI 1640 media (Mediatech, Manassas, VA, USA) supplemented with 5% fetal bovine serum (FBS) (Atlanta Biologicals, Lawrenceville, GA, USA), 5% bovine serum (Life Technologies, Auckland, New Zealand), 0.01 mg/mL of human insulin (Roche, Indianapolis, IN, USA), 10 mM HEPES (Corning, Corning, NY, USA), 100 IU penicillin (Mediatech), 100 μg/mL streptomycin (Mediatech), 2 mM L-Alanyl-L-Glutamine (Glutagro^TM^, Corning), and 1 mM sodium pyruvate (Corning). The cells were incubated at 37 °C in a humidified incubator with 5% CO_2_. The drugs used in this study include the following: auranofin (Sigma Chemical Co., St. Louis, MO, USA), bortezomib (BZ) (Velcade^®^, Millennium Pharmaceuticals, Cambridge, MA, USA), L-buthionine sulfoximine (L-BSO) (Sigma), and N-acetyl cysteine (NAC; Sigma). 

### 2.2. Cellular Vitality 

To determine the wellbeing of the cells, we performed a cellular vitality assay [40] evaluating mitochondrial enzyme activities as surrogate markers of drug toxicity in control conditions versus drug treatment. PEO1 and PEO4 cells growing at 70% confluency were harvested and seeded in triplicate in 96-well plates at a density of 2.5 × 10^3^ cells/well in HGSOC medium and allowed to adhere overnight at 37 °C in 5% CO_2_. The cells were then treated with varying concentrations of auranofin for 72 h. Cell vitality was measured by adding 10 μL/well of 5 mg/mL MTT [3-(4,5-dimethylthiazol-2-yl)-2,5-diphenyltetrazolium bromide] (Life Technologies, Burlington, ON, Canada) in PBS solution. The cells were incubated for 4 h at 37 °C in 5% CO_2_ where the tetrazolium dye was reduced to insoluble formazan. One hundred microliters/well of 10% sodium dodecyl sulfate (SDS)/0.01 M HCl was added to stop the reaction. The absorbance was recorded at 570 nm following overnight incubation. Blank controls were subtracted and the percentage of cell vitality relative to the control was calculated.

### 2.3. Cellular Viability

Clinically platinum-sensitive (PEO1) and platinum-resistant (PEO4) HGSOC cells were treated with increasing concentrations of auranofin for 72 h. The cells were collected, centrifuged, and the remaining cell pellet was resuspended in 1 mL of cell culture medium. An aliquot of cells was taken and stained with the Muse^®^ count and viability reagent (Luminex, Austin, TX, USA) for 5 min; this reagent contains a DNA-binding dye that tags nucleated cells, and a second dye that differentiates live from dead cells by penetrating cells with compromised membrane integrity (i.e., non-viable cells). Stained cells were analyzed using the Muse^®^ micro-capillary cytometer (Millipore, Hayward, ON, Canada), and the viable cell number and total cell number were determined. 

### 2.4. Clonogenic Survival

To assess the residual toxicity of auranofin on HGSOC cells, 1000 viable cells were taken from the cell culture treated with auranofin for 72 h and plated in media devoid of drugs in a 6-well plate for 2 weeks. The experiment was terminated once the vehicle-treated group contained positive colonies. Positive colonies refer to colonies that contain 50 or more cells; this is used as a measure of assessing the proliferative capacity of the cells in a long-term period even though they survived the initial 72 h of drug treatment; in other words, we tested how the exposure to auranofin affected their long-term proliferative capacity.

### 2.5. Cell Cycle Distribution 

Following the 72 h treatment with auranofin, PEO1 and PEO4 cells were fixed using 4% paraformaldehyde (PFA) and stored at 4 °C overnight. Samples were centrifuged and the cell pellet was washed with 500 µL of 1× phosphate-buffered saline (PBS) (Corning, Manassas, VA, USA). Two hundred thousand cells were collected and centrifuged at 2000× *g* for 5 min. The supernatant was discarded, and the pellet was resuspended in 200 μL of cell cycle buffer containing 0.5 mg/mL propidium iodide, a cell-permeable DNA intercalating agent that serves to analyze the status of DNA content. Cell cycle analysis was completed using the Muse^®^ micro-capillary cytometer (Millipore). This method was previously described in detail [15]. 

### 2.6. Protein Lysate Preparation and Western Blot Analysis

PEO1 and PEO4 cells were treated with increasing concentrations of auranofin for 72 h, and whole cellular extracts were collected at the end of the incubation. The cells were centrifuged at 1500× *g* for 6 min, the supernatant was removed, and the cell pellet was resuspended in 1 mL of cold 1× PBS. The samples were centrifuged at 2000× *g* for an additional 5 min, the supernatant was removed, and the cell pellets were snap frozen in liquid nitrogen and stored at −80 °C until further processing. The proteins were isolated by first adding lysis buffer to the cell pellets. The lysis buffer was prepared as follows: 0.5% NP-40, 1 mM dithiothreitol (DTT), 1 mM phenylmethylsulphonyl fluoride (PMSF), 2 µg/mL aprotinin, 2 µg/mL pepstatin, 2 µg/mL leupeptin, 50 mM sodium fluoride, and 1 mM sodium orthovanadate. The cell pellets were resuspended in the lysis buffer by gentle vortexing, and were placed on ice on a shaker for 30 min at 4 °C. The samples were centrifuged at 12,000× *g* for 15 min at 4 °C. The proteins in the supernatant were collected and transferred to separate tubes. Protein samples were then quantified using the Pierce BCA Protein colorimetric assay purchased from Thermo Fisher Scientific (Rockford, IL, USA), and absorbance was measured at 562 nanometers using the Bio-Tek Cytation 3 Multi-Mode Reader (Agilent, Santa Clara, CA, USA). The proteins were electrophoresed on 10% SDS-polyacrylamide gels. Following the transfer onto the PVDF membranes, the membranes were blocked in 5% non-fat dry milk at room temperature for 1 h and incubated with the primary antibodies at 4 °C overnight. The membranes were washed 5 times in 1× TBS-T for 5 min each and incubated with the secondary antibody for 1 h. The secondary antibody was removed, and the membranes were washed again 5 times in 1× TBS-T for 5 min each. The membranes were then imaged using the Bio-Rad ChemiDoc Touch Imaging System (Bio-Rad, Hercules, CA, USA). The primary antibodies used were monoclonal anti-β-actin produced in mice as clone AC-15 (A5442, Sigma), polyclonal anti-PARP produced in rabbit (9541, Cell Signalling Technology, Danvers, MA, USA), and polyclonal anti-ubiquitin produced in rabbit (3933S, Cell Signalling). Secondary antibodies were goat anti-rabbit IgG (H + L) conjugate (1706515, BioRad) and goat anti-mouse IgG (H + L)-HRP conjugate (1706516, BioRad). 

### 2.7. Detection of DNA Damage 

To determine whether auranofin induces DNA damage in HGSOC, PEO1 and PEO4 cells were treated with 1, 2, or 4 µM auranofin for 72 h. The cells were collected and centrifuged at 300× *g* for 5 min, and the supernatant was removed. The cells were resuspended in 50 µL of 1× assay buffer per 100,000 cells, and equal volume of fixation buffer was added to the cells. The samples were incubated on ice for 10 min, spun down at 300× *g* for 5 min, and the supernatant was discarded. The cells were resuspended in 90 µL of 1× assay buffer for every 100,000 cells. Cells were then stained with 10 µL of antibody working solution that was prepared by combining 5 µL of anti-phospho-ATM (Ser1981) labelled with phycoerythrin (PE), and 5 µL of anti-phospho-histone H2A.X (Ser139) labelled with PE-Cyanine^®^5 (PeCy5). The samples were incubated at room temperature for 30 min protected from light. One hundred microliters of 1× assay buffer were added, and samples were centrifuged at 300× *g* for 5 min. The supernatant was discarded, and the cells were resuspended in 200 µL of 1× assay buffer. Cells were analyzed using the multicolor DNA damage protocol (Luminex) with the Guava Muse^®^ cell analyzer (Millipore).

### 2.8. Detection of Annexin-V Binding 

PEO1 and PEO4 cells were treated with 2 or 4 µM auranofin for 72 h. The treated cells were collected and resuspended in different volumes of media to obtain 1 × 10^5^ to 5 × 10^5^ cells per mL. A 100 µL cell suspension containing approximately 1 × 10^6^ cells was placed in a tube, and 100 µL of the annexin V and dead cell reagent (Luminex) was added for 20 min at room temperature in the dark. Annexin V is a calcium-dependent phospholipid binding protein that binds to phosphatidylserine (PS), which translocates to the extracellular surface of the plasma membrane during early apoptosis. The dead cell reagent differentiates live and dead cells, by integrating into the membrane of late apoptotic and dead cells owing to the loss of membrane structural integrity. The cells were analyzed using the annexin V and dead cell protocol in the Guava Muse^®^ cell analyzer (Millipore).

### 2.9. Measurement of Caspase-3/7 Activation 

PEO1 and PEO4 cells treated with 2 or 4 µM auranofin for 48 h were collected. The Muse caspase-3/7 kit (Luminex) was used. A 50 µL suspension containing approximately 5 × 10^5^ cells was placed in a tube. Five microliters of the caspase-3/7 reagent working solution, prepared by diluting the caspase-3/7 stock 1:8 with 1× PBS, were added to the cells and incubated in the dark for 30 min in a 37 °C, 5% CO_2_ incubator. This reagent binds to a DNA-binding DEVD peptide substrate that, upon activation of caspase-3/7, is cleaved and then translocates to the nucleus to bind DNA and emit fluorescence. Cells were then stained for 5 min at room temperature in the dark with 150 µL of Muse caspase-7-AAD substrate working solution prepared at a 1:75 dilution using 1× assay buffer. 7-AAD is a cell-permeable DNA-binding dye that integrates into cells that have lost their membrane structural integrity. The analysis was performed using the caspase-3/7 protocol on the Guava Muse^®^ cell analyzer (Millipore). 

### 2.10. Treatment with a Caspase Inhibitor

PEO1 and PEO4 cells were pre-treated with 50 µM z-DEVD-fmk for 2 h (Selleck Chemicals, Houston, TX, USA). This is a specific irreversible caspase-3 inhibitor that also potently inhibits caspase-6, caspase-7, caspase-8, and caspase-10. A concentration of 2 or 4 µM auranofin was added for 24 h to PEO1 cells, and for 48 h to PEO4 cells, and cell viability was assessed by cytometry and analyzed using the Guava Muse^®^ cell analyzer (Millipore). 

### 2.11. Detection of Mitochondrial Membrane Depolarization 

PEO1 and PEO4 cells treated with 2 or 4 µM of auranofin for 24 h were collected. The cells were resuspended in 500 µL of 1× assay buffer for a final concentration of 5 × 10^5^ cells per mL. One hundred microliters of the cell suspension were placed in a 1.5 mL centrifuge tube and were incubated for 20 min at 37 °C 5% CO_2_ with 95 µL of Mito-Potential working solution prepared by diluting the Muse Mito-Potential dye at 1:1000 in 1× assay buffer. Five microliters of the Muse Mito-Potential 7-AAD reagent (Luminex) were added to each tube and incubated for 5 min at room temperature. The analysis was performed using the Mito-Potential protocol on the Guava Muse^®^ cell analyzer (Millipore). 

### 2.12. Assessment of Intracellular ROS Levels 

To assess whether auranofin stimulates the production of ROS in HGSOC, PEO1 and PEO4 cells were treated with 8 µM AF for 4 h. Intracellular superoxide levels were measured using an oxidative stress assay (Luminex). This assay uses the cell-permeable reagent dihydroethidium (DHE), which binds to DNA and produces red fluorescence upon interaction with superoxide ions. Following treatment, the cells were collected and prepared in 1× assay buffer at 1 × 10^6^ to 1 × 10^7^ cells per mL. An intermediate solution of the Muse oxidative stress reagent was prepared by diluting it to 1:100 with 1× assay buffer. To prepare the Muse oxidative stress working solution, the intermediate solution was diluted to 1:80. One hundred and ninety microliters of the Muse oxidative stress working solution were added to 10 µL of cells and mixed thoroughly by pipetting up and down. The samples were incubated for 30 min at 37 °C. The stained samples were analyzed using the oxidative stress protocol on the Muse^®^ cell analyzer (Millipore). 

### 2.13. Drug Interaction between Auranofin and L-BSO 

Two hundred thousand PEO1 and PEO4 cells were plated per well in 6-well plates and allowed to attach overnight. The drug interaction was studied in triplicate experiments using two doses of auranofin with a fixed dose of L-BSO: 2 or 4 µM auranofin with or without 5 µM of L-BSO for 72 h. Upon treatment, the cells were collected, and viability and cell number were measured using the Muse^®^ count and viability reagent (Luminex). The combination index (CI) was then calculated using the method of Chou and Talalay [41] utilizing the CompuSyn Software Version 1.0 (ComboSyn Inc., Paramus, NJ, USA). For a specific drug combination, a CI > 1 was considered antagonistic, CI = 0 indicated no drug interaction, CI = 1 indicated additivism, and CI < 1 denoted synergism. 

### 2.14. Measurement of TrxR Activity 

We used a thioredoxin reductase (TrxR) assay kit purchased from Abcam (Cambridge, MA, USA). In this colorimetric assay, TrxR activity was measured by the reduction of 5,5′-dithiobis (2-nitrobenzoic) acid (DTNB) using NADPH to 5-thio-2-nitrobenzoic acid (TNB^2−^), and absorbance was measured at 412 nanometers. Two million PEO1 or PEO4 cells were treated with 1, 2, or 4 µM auranofin for 24 h. The cells were placed on ice, collected by scraping, and washed twice with 1× PBS; they were centrifuged at 1500× *g* for 6 min, and the supernatant was removed. The cells were resuspended again in 1 mL of 1× PBS and centrifuged at 2000× *g* for 5 min. The supernatant was decanted, and the cell pellet was snap frozen in liquid nitrogen and stored at −80 °C until further processing. The cell pellet was homogenized on ice with 150 µL of cold assay buffer containing 1× protease inhibitor cocktail (Abcam), and centrifuged at 12,000× *g* for 15 min at 4 °C. The protein concentration in the supernatant was quantified using the BCA protein assay (Pierce). Two sets of 50 µg of protein for each sample and 10 µL of the TrxR positive control were loaded into a 96 well plate, and the volume was adjusted to 50 µL with TrxR assay buffer. Ten microliters of TrxR inhibitor were added to one set to test the background enzyme activity, and 10 µL of assay buffer was added to the other set to measure total DTNB reduction. A standard curve was generated with 0, 10, 20, 30, 40, and 50 nmol/well, which was adjusted to a final volume of 100 µL with assay buffer. A reaction mix containing TrxR assay buffer, DTNB solution, and NADPH was prepared, and 40 µL of the reaction mix was added to the positive control and to each sample and mixed well. Optical density (OD) was measured at 412 nanometers (nm) to obtain A_1AB_ and A_1INH_, and the samples were incubated for 20 min at 25 °C and measured at 412 nm again to obtain A_2AB_ and A_2INH_. To determine the optical density of TNB^2-^ generated by TrxR, the following calculation was used: ∆A_412nm_ = (A_2AB_ − A_2INH_) − (A_1AB_ − A_1INH_); where AB is assay buffer and INH is the inhibitor. TrxR activity was determined using the following formula: TrxR activity = ∆B/[(T2 − T1) × V] × sample dilution factor = nmol/min/mL = mU/mL; ∆B: nmol was calculated by applying ∆A_412 nm_ to the TNB standard curve; T_1:_ time of the first reading (min); T_2:_ time of the second reading (min); and V: pretreated sample volume (mL). One unit of TrxR is the amount of enzyme that generates 1.0 µmol of TNB per minute at 25 °C.

### 2.15. In Vitro Analysis of Total GSH

The GSH assay kit was purchased from Abcam. This colorimetric assay measures the concentration of reduced GSH in vitro. The kit contains a chromophore, and the reduction of the chromophore by an enzyme can be determined kinetically by measuring the absorbance at 450 nanometers. The absorbance is directly proportional to the amount of GSH that is present in each sample. PEO1 and PEO4 cells were treated with 1 or 2 µM auranofin in the presence or absence of 5 µM L-BSO for 24 h. Cells were placed on ice, collected by scraping, and washed twice with 1× PBS. Cells were centrifuged at 1500× *g* for 6 min, and the supernatants were removed. Cells were resuspended again in 1 mL of 1× PBS, split into two tubes to obtain two sets of samples, and centrifuged at 2000× *g* for 5 min. The supernatants were decanted, and the cell pellets were snap frozen in liquid nitrogen and stored at −80 °C until further processing. One set of samples was used to determine the protein concentration in mg/mL. The other set of samples was homogenized on ice using 100 µL of 5% sulfosalicylic acid, vortexed, and kept on ice for 10 min. The samples were then centrifuged at 12,000× *g* at 4 °C for 20 min, and the supernatant was collected and kept on ice. The samples were diluted 5-fold using the GSH assay buffer, and 10 µL of the diluted samples was added per well in a 96-well plate for the sample well and the sample background control well. The volume of each sample was adjusted to 20 µL/well with GSH assay buffer. A standard curve was produced with 0, 0.4, 0.8, 1.2, 1.6, and 2 nmol/well of the GSH standard, which was adjusted to 20 µL/well with GSH assay buffer. A reaction mix containing substrate mix A, diluted enzyme mix A, enzyme mix B, enzyme mix C, and substrate mix B was prepared; 80 µL of the reaction mix was added to each sample well and the GSH standard wells. A background control mix was prepared containing everything except the diluted enzyme mix A, and 80 µL of the background control mix was added to the wells of the sample background controls. The absorbance was then measured kinetically at 450 nanometers for 60 min at room temperature, and the absorbances at two time-points within the linear range were selected for each sample. The concentration of GSH was then determined using a formula recommended by the provider as follows: first, the rate of each standard reading and sample reading was calculated; rate = [(OD_2_ − OD_1_)/t_2_ − t_1_)], where OD_2_ is the optical density at the second time point, and OD_1_ is the optical density for the first time point; t_1_ is the initial time in min, and t_2_ is the second time in min. The 0-standard rate was subtracted from all the standard rates, and the GSH standard curve was plotted to obtain the slope of the curve; thereafter, the rate of the background-corrected samples was calculated by subtracting the sample background control rate from the sample rate; rate of the background-corrected samples = [rate _sample_ − rate _background control_]; then, the rate of the background-corrected samples was applied to the GSH standard curve to calculate the amount of GSH in each sample: B = rate background-corrected samples/slope of the standard curve; and, finally, the GSH amount in sample was calculated as (B/[V × P]) × D = nmol/mg, where B is the amount of GSH from the standard curve (nmol), V is the volume of sample added in each well (mL), P is the protein concentration in mg/mL, and D is the sample dilution factor. 

### 2.16. Statistics

For tests involving Western blot analysis, the experiments were repeated at least twice with a similar outcome. All other data represent triplicate experiments and are expressed as the mean ± SEM. Differences were considered statistically significant if *p* < 0.05. GraphPad Prism 9 (GraphPad Software, La Jolla, CA, USA) was used for statistical analysis of data using *t*-test to compare two groups, or one-way ANOVA followed by Tukey’s multiple comparison test to compare more than two groups within an experiment.

## 3. Results

### 3.1. Auranofin Reduces the Vitality of HGSOC Cells Regardless of Their Sensitivities to Cisplatin 

**Figure 1:**
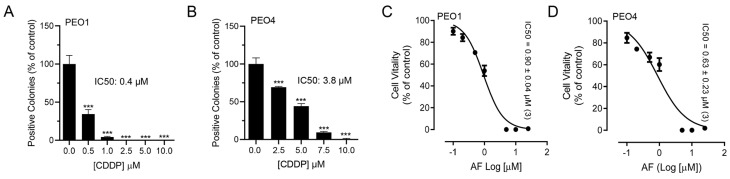
Effect of auranofin (AF) on the vitality of PEO1 or PEO4 cells. Cells were treated with DMSO (vehicle) or with various concentrations of cisplatin (CDDP) or auranofin (AF) for 72 h. At the end of the treatment, the cells were subjected to a clonogenic survival assay (for CDDP) and vitality assay (for AF), as detailed in Materials and Methods. Panels (**A**,**B**) show contrasting clonogenic survival among the cell lines in response to CDDP whereas panels (**C**,**D**) show a similar decrease in vitality caused by AF in the two cell lines. *** *p* < 0.01 compared to 0.

To test whether blocking TrxR impairs the metabolic activity or wellbeing of ovarian cancer cells, we exposed sibling cell lines to auranofin and assessed their vitality by measuring the activity of mitochondrial enzymes. We used a pair of cell lines, termed PEO1 and PEO4, which have different sensitivities to platinum. For instance, we recently demonstrated that PEO4 cells are approximately ten times less sensitive to cisplatin than their PEO1 siblings obtained from the same patient earlier during disease evolution [15]. Despite the large difference in the platinum sensitivity between the two cell lines (Figure 1A,B), both cell types responded to the increased concentrations of auranofin with a similar impairment in wellbeing as denoted by the similar decrease in their vitality demonstrated by the IC50s, which were similar in both cell lines (Figure 1C,D; see actual IC50s on the right corner of the panels). This signifies that both cell lines are equally sensitive to auranofin, at least in terms of the impairment of their mitochondrial metabolic activities. 

### 3.2. Auranofin Inhibits TrxR Activity 

**Figure 2:**
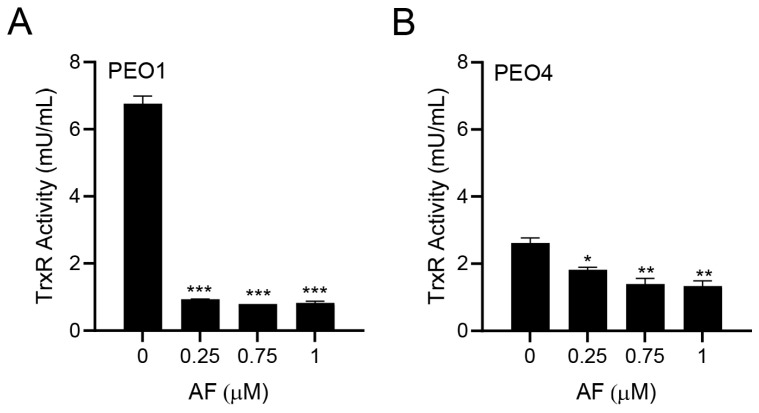
Effect of auranofin (AF) on the activity of the enzyme TrxR. In this colorimetric assay, TrxR activity was measured by the reduction of 5,5′-dithiobis (2-nitrobenzoic) acid (DTNB) using NADPH, to 5-thio-2-nitrobenzoic acid (TNB^2−^) as detailed in Materials and Methods. * *p* < 0.05, ** *p* < 0.01, and *** *p* < 0.001 when compared to vehicle. (**A**); PEO1 cells. (**B**); PEO4 cells.

Since auranofin is known to inhibit TrxR [33], we tested whether this indeed occurred in PEO1 and PEO4 cells. The PEO1 cells had a relatively high basal TrxR activity, which was comparable to that of the rat liver homogenate used as a positive control. Once treated with different concentrations of auranofin for 24 h, there was a potent inhibition of the TrxR activity (Figure 2A). In the PEO4 cells, the basal TrxR enzymatic activity was lower than that found in the PEO1 cells; nevertheless, the activity of TrxR was significantly reduced further by auranofin in a concentration-dependent manner (Figure 2B). Our results demonstrate that in HGSOC cells, auranofin hinders the activity of its primary target, TrxR. 

### 3.3. Auranofin Triggers the Accumulation of Reactive Oxygen Species 

**Figure 3:**
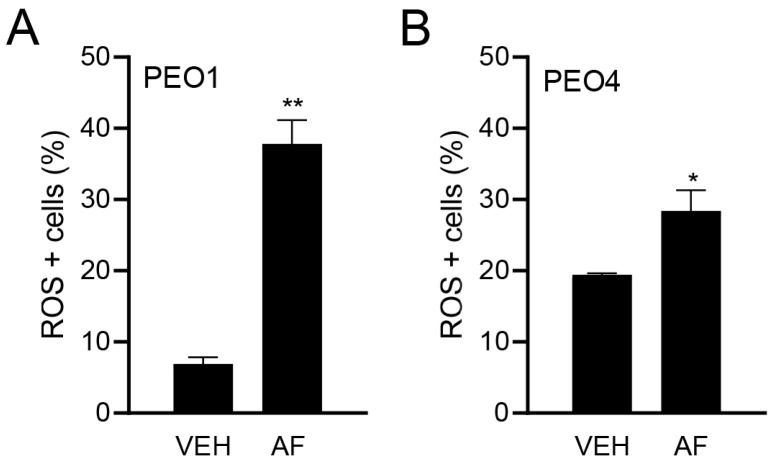
Effect of vehicle (VEH) or auranofin (AF) on the production of ROS. The oxidative stress method measures the levels of a cell-permeable reagent named dihydroethidium (DHE), which upon interaction with superoxide, binds to DNA and produces red fluorescence. Treatment was performed with 8 μM AF for 4 h. * *p* < 0.05 and ** *p* < 0.01 compared to vehicle. (**A**); PEO1 cells. (**B**); PEO4 cells.

Although the TrxR activity was assessed, a direct measure of the influence of TrxR inhibition is the causation of oxidative stress. Thus, to confirm the effect of auranofin on oxidative stress, we measured the ROS production in HGSOC cells in the presence or absence of auranofin for 4 h. Figure 3A,B show the significant increase in the percentage of ROS-positive cells in response to auranofin in both the platinum-sensitive PEO1 cells and platinum-resistant PEO4 cells. 

### 3.4. Auranofin Kills HGSOC Cells in Association with Induction of Apoptosis 

**Figure 4:**
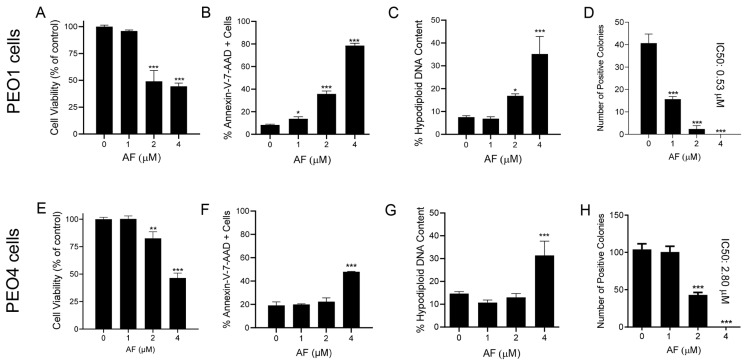
Viability of PEO1 and PEO4 cells after 72 h of treatment with auranofin (AF) assessed using a cytometric viability assay (**A**,**E**). In a similar experiment, cells were stained with Annexin V and 7-AAD to determine apoptosis (**B**,**F**). Cells remaining from the viability experiment were also stained with propidium iodide and studied for cell cycle distribution; only the hypodiploid DNA content is shown (**C**,**G**). Finally, cells that were still alive after 72 h of treatment with AF shown in (**A**,**E**), were subjected to a clonogenic survival assay to define their long-term reproductive capacity (**D**,**H**). * *p* < 0.05, ** *p* < 0.01 and *** *p* < 0.001 compared with cells treated with vehicle.

The reduction in the vitality of cells exposed to auranofin shown in Figure 1 suggests that the drug may have cytotoxic effects. Indeed, auranofin reduced the cell viability (Figure 4A,E), which was associated with an increase in markers of apoptotic cell death, such as the double labelling of Annexin-V and 7-AAD (Figure 4B,F). Confirmation of apoptosis induced by auranofin was elicited by the accumulation of cells with hypodiploid DNA content (Figure 4C,G). Finally, if the cells that were still viable after 72 h of treatment (Figure 4A,E) were placed in a clonogenic survival plate in the absence of auranofin, the long-term toxicity of the previous exposure to the drug was clearly depicted by the concentration-dependent reduction of viable colonies (Figure 4D,H). In contrast to the effects on vitality, in which both the platinum-sensitive and resistant cells responded to auranofin in a similar fashion, measuring the lethality-related parameters clearly showed that auranofin is more potent against the PEO1 cells than against the PEO4 cells. The clonogenic survival depicts a five-fold difference, with an IC50 of 0.53 μM for PEO1 cells (Figure 4D) and an IC50 of 2.8 μM for PEO4 cells (Figure 4H). 

### 3.5. Auranofin Induces Dissipation of the Mitochondrial Potential, a Phenomenon That Is Prevented by the Presence of the ROS Scavenger N-acetyl Cysteine 

**Figure 5:**
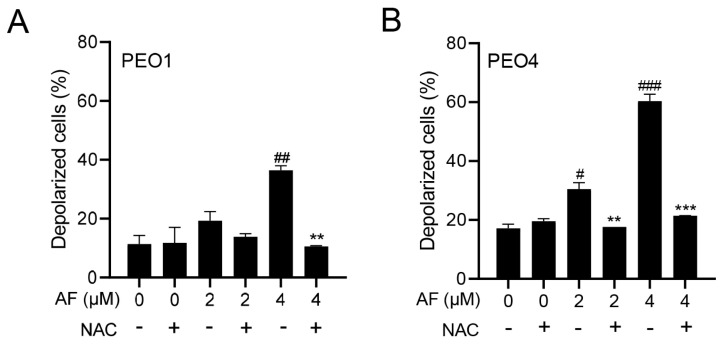
Auranofin (AF) increases the percentage of cells with depolarized mitochondria in a dose—dependent fashion. # *p* < 0.05, ## *p* < 0.01, and ### *p* <0.001 when compared to vehicle–treated controls; the addition of NAC blocked the effect; ** *p* < 0.01 and *** *p* < 0.001 when compared to the corresponding AF-treated groups. Mitochondrial depolarization was assessed by a cytometric method that utilizes a Mito—Potential dye. A high membrane potential drives the dye into the inner membrane of intact mitochondria, resulting in high fluorescence. Cells with depolarized mitochondria showed decreased fluorescence. NAC, N-acetyl cysteine. (**A**) PEO1 cells. (**B**) PEO4 cells.

The cellular energy produced during mitochondrial respiration is stored as an electrochemical gradient across the mitochondrial membrane, and this accumulation of energy in healthy cells creates a mitochondrial transmembrane potential (ΔΨ_m_) that enables the cells to drive the synthesis of ATP. The collapse of this potential is believed to coincide with the opening of the mitochondrial permeability transition pores, leading to the release of cytochrome C into the cytosol, which then triggers the downstream events in the apoptotic cascade [42,43]. Thus, the depolarization of the inner mitochondrial membrane potential is a reliable indicator of mitochondrial dysfunction and cellular death by apoptosis [44]. Here, we show that the treatment of the PEO1 or PEO4 cells with auranofin caused the loss of ΔΨ_m_ and further illustrated that the presence of the ROS scavenger N-acetyl cysteine (NAC) [45] prevented the depolarization of the mitochondrial membrane (Figure 5A,B). 

### 3.6. Auranofin-Induced Cell Death Is Prevented by N-acetyl Cysteine 

**Figure 6:**
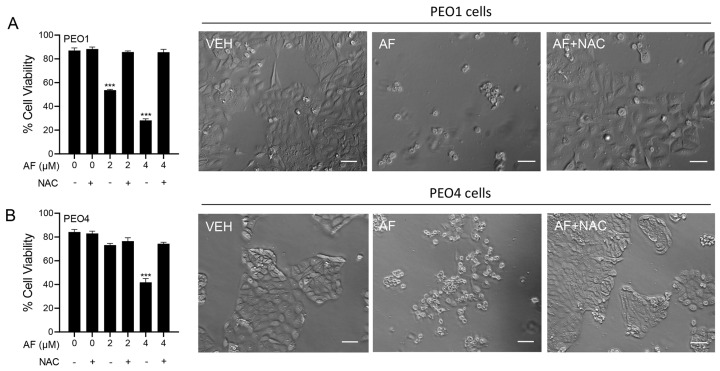
PEO1 (**A**) or PEO4 cells (**B**) were treated with auranofin (AF) in the absence or presence of 5 mM NAC. Cell viability was assessed after 72 h using a microcytometer. *** *p* < 0.001 vs. AF + NAC. In the right panels, phase−contrast images were obtained after 72 h of incubation with the indicated drugs. VEH, vehicle; AF, auranofin; NAC, N-acetyl cysteine. Scale bars = 50 μm.

To determine whether the mechanism of cytotoxicity by auranofin is dependent on the production of ROS, the PEO1 and PEO4 cells were cultured in the presence of 2 or 4 μM auranofin with or without the addition of 5 mM NAC. The results presented in Figure 6 show that auranofin reduced the cell viability in a concentration-related manner and this effect was prevented by the antioxidant NAC (left panels in Figure 6). The right panels in Figure 6 clearly show the morphological deterioration of the cell cultures in the presence of auranofin and how the deterioration was prevented by NAC. 

### 3.7. NAC Prevented Auranofin-Induced Caspase-3/7 Activation, Cleavage of PARP, and Induction of γH2AX 

**Figure 7:**
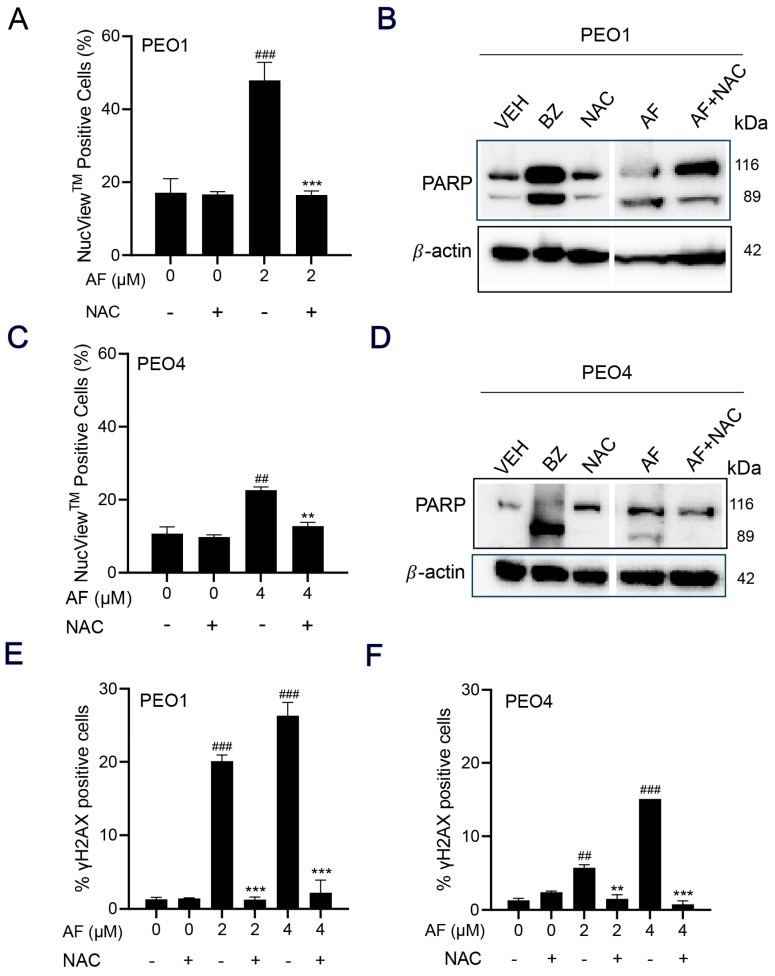
PEO1 (**A**) or PEO4 cells (**C**) were treated with auranofin (AF) in the absence or presence of 5 mM NAC for 24 h (to measure PARP), 48 h (to measure caspase−3/7 activity), or 72 h (to measure accumulation of γH2AX). In (**A**,**C**), cells were exposed to the Muse^®^ Caspase−3/7 reagent, which is cell membrane−permeable, in combination with a dead cell dye (7-AAD). ## *p* < 0.01 and ### *p* < 0.001 compared to vehicle. ** *p* < 0.01 and *** *p* < 0.001 compared to AF. (**B**,**D**) depict the effect of AF and NAC on the cleavage of PARP as detected by Western blotting. In this experiment, cells treated with 20 nM bortezomib (BZ) were used as a positive control of PARP cleavage. (**E**,**F**) show the accumulation of γH2AX in response to AF with and without NAC. ## *p* < 0.01 and ### *p* < 0.01 compared to vehicle. ** *p* < 0.01 and *** *p* < 0.001 compared to the respective concentration of AF. The original image of Western blots are shown in the Figure S3.

A caspase-3/7 cytometric assay was utilized to quantify the activation of executer caspases in response to auranofin. The results clearly indicate that auranofin was more effective in the PEO1 than in the PEO4 cells; nonetheless, such activation was prevented by the presence of NAC (Figure 7A,C). Likewise, and in both cell lines, when assessing the induction of apoptosis by measuring the cleavage of PARP, we observed that auranofin was effective in inducing such a cleavage, which was, at least in part, prevented by NAC (Figure 7B,D). Finally, auranofin triggered the accumulation of the DNA damage marker γH2AX in both the PEO1 (Figure 7E) and PEO4 (Figure 7F) cells and this was entirely abrogated by NAC. These results suggest that auranofin mediates both executer caspase activation and DNA damage by the generation of reactive oxygen species (ROS). 

### 3.8. The Cytotoxic Effect of Auranofin and L-BSO against HGSOC Is Synergistic and Associates with Enhanced ROS Production and Reduced Levels of GSH 

**Figure 8:**
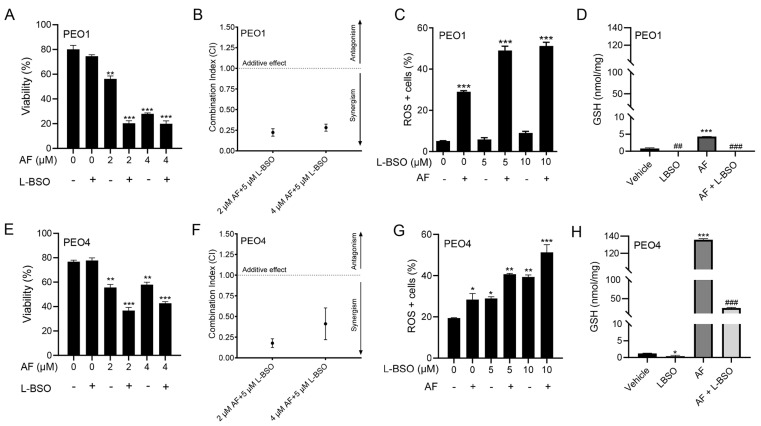
PEO1 (**A**) or PEO4 (**E**) cells were treated with 2 or 4 μM auranofin (AF) for 72 h in the presence (+) or absence (−) of L-buthionine sulfoximine (L-BSO), and viability was recorded by cytometry. (**B**,**F**) The combination indexes of the drug interaction using different combinations of AF and L-BSO showed a CI < 1, indicating synergism between the drugs. The CIs were calculated using the viability data of cells treated with the specified concentrations of AF and/or L-BSO in three independent experiments. (**C**,**G**) show ROS levels in response to AF with (+) or without (−) L-BSO. (**D**,**H**) display GSH levels, respectively, in PEO1 cells or in PEO4 cells treated with vehicle, 2 μM AF, or the combination of 2 μM AF and 5 μM L-BSO. In (**A**,**C**,**E**,**G**), * *p* < 0.05, ** *p* < 0.01 and *** *p* < 0.001 vs. control. In (**D**,**H**), *** *p* < 0.001 vs. vehicle, ## *p* < 0.01 vs. vehicle, and ### *p* < 0.001 vs. AF.

We hypothesized that the toxicity of auranofin could be augmented by blocking an additional, likely compensatory, antioxidant system: GSH. Thus, we decided to simultaneously block TrxR with auranofin and GSH with L-BSO [46]. Figure 2 and Figure 4 show that auranofin blunted TrxR activity and caused cell death. Figure 8 documents that the combination of auranofin and L-BSO leads to a further reduction in the viability when compared to auranofin alone (Figure 8A,E). The interaction between auranofin and L-BSO was synergistic based on the CI method of Chow and Talalay (Figure 8B,F). Furthermore, the elevation of ROS induced by auranofin in both cell types was significantly increased in combination with L-BSO (Figure 8C,G). Finally, we showed that blocking TrxR by auranofin alone leads to a compensatory increase in GSH, which is significantly reduced by the presence of L-BSO (Figure 8D,H). 

### 3.9. The Lethal Effect of Auranofin and L-BSO against HGSOC Is Prevented by the Presence of the ROS Scavenger NAC 

**Figure 9:**
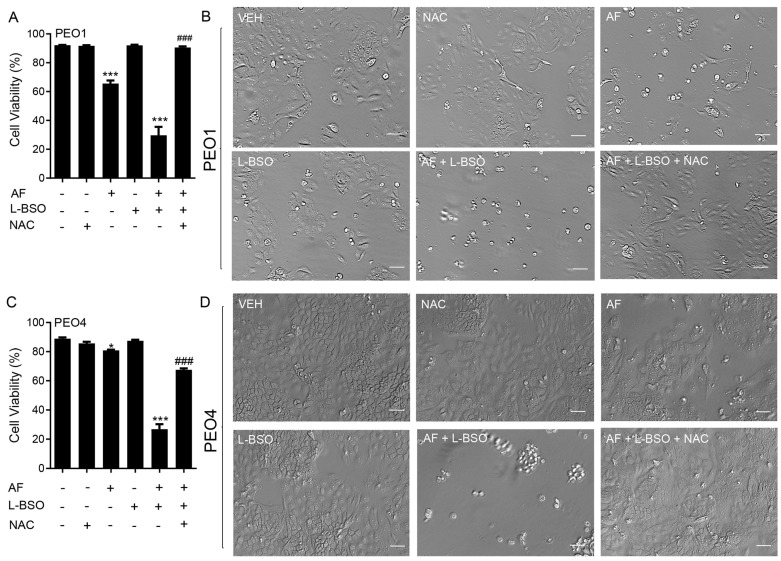
Cell viability (**A**,**C**) and phase−contrast images (**B**,**D**) of PEO1 and PEO4 cells receiving vehicle (VEH), N-acetyl cysteine (NAC; 5 mM), auranofin (AF; 2 μM), L-buthionine sulfoximine (L-BSO; 5 μM), or the combination of AF/L-BSO or AF/L-BSO/NAC. * *p* < 0.05 and *** *p* < 0.001 compared to vehicle; ### *p* < 0.001 compared to the AF/L-BSO group. Scale bars = 50 μm.

We investigated whether the cytotoxicity of auranofin in combination with L-BSO was prevented by the ROS scavenger and antioxidant NAC. In Figure 9A for PEO1 cells and Figure 9C for PEO4 cells, we show that the cell death caused by 2 μM auranofin was greatly enhanced by the presence of 5 μM L-BSO. Of interest, such a reduction in the cell viability was almost totally reversed by the presence of NAC. The phase-contrast panels in Figure 9B,D show that the morphology and growth of the cells were negatively affected by auranofin alone and further impaired by auranofin in combination with L-BSO. Consistent with the viability data shown in panels A and C, the presence of NAC prevented the cytotoxic effects, and the cell cultures resemble that of the vehicle-treated controls.

## 4. Discussion

The cytotoxic properties of auranofin as a monotherapy or in combination with other drugs have been studied in various cancers, including lung [47,48,49,50], breast [51,52,53], pancreatic adenocarcinoma [36], colorectal [54], gastric [55], mesothelioma [34], melanoma [56], malignant B-cells, and acute lymphoblastic leukaemia (ALL) [57,58,59]. In ovarian cancer, auranofin has been shown to block the growth of A2780, SKOV-3, and IGROV-1 cells [17,20,24,38,60], but none of these cell lines represent the most common histotype which is HGSOC [19,61]. In our study, we used two cell types, PEO1 (platinum sensitive) and PEO4 (platinum resistant), which were isolated from the same patient throughout the course of the disease [27] and genotypically identified as HGSOC [28]. These provide a valuable model of cellular changes during disease progression, in which PEO1 cells contain a *BRCA2* germline mutation, whereas PEO4 cells have a restored version of the gene [28,62]. One previous study reported that PEO4 cells were resistant to the combination of auranofin with HSP90 inhibitors [63], but our results showed cytotoxicity by auranofin alone or in combination with the GSH inhibitor L-BSO against both the platinum-sensitive PEO1 cells and their sibling platinum-resistant PEO4 cells. 

Auranofin would be particularly advantageous to improve the therapy for HGSOC because the gold complex has been already approved by the FDA against rheumatoid arthritis, and it is currently enrolled in several clinical trials as a monotherapy and in combination with other drugs (reviewed in [33]). This indicates the feasibility of repurposing auranofin against HGSOC as it has been shown to be clinically tolerable. 

The effect of auranofin on cellular vitality or wellbeing [40] assessed through the surrogate activation of mitochondrial enzymes, revealed that both PEO1 and PEO4 cells were equally affected by the drug suggesting that there is no cross-resistance between the platinum agent and the gold complex. However, when we studied the viability (i.e., the capacity of auranofin to kill the cancer cells), we observed that the drug was more effective against the PEO1 cells than the PEO4 cells, suggesting that in terms of lethality, there is some degree of cross-resistance between platinum and auranofin. This was supported by a series of additional studies on the short-term cytotoxic responses to stress in which we showed that the PEO4 cells were again less sensitive to the drug than the PEO1 cells. 

The induction of apoptosis/death by auranofin represented by the positive staining of Annexin V and 7-AAD was greater in the PEO1 cells than in the PEO4 cells. The induction of apoptosis has been documented as one of the principal short-term cytotoxic effects of auranofin in other malignancies, including multiple myeloma, acute myeloid leukemia, murine triple-negative breast cancer, lung cancer, and mesothelioma [34,47,57,64]. We further confirmed the induction of apoptosis with the accumulation of hypo-diploid DNA during exposure to auranofin, which concurs with similar effects reported in non-small cell lung carcinoma (NSCLC) cells [65]. The colony formation assay showed that even when the plasma membrane permeability was not altered in the short term, auranofin caused the long-term inhibition of cellular reproduction by inhibiting their clonogenic survival, which was again more pronounced (~five-fold) in the PEO1 cells than in the PEO4 cells. The inhibition of positive colony formation by auranofin has also been demonstrated in a stem-like cancer cell side population and in SKOV-3 epithelial ovarian cancer cells in a p53-independent manner [23,66]. 

When we explored the direct effect of auranofin against TrxR activity, the presumed target of the drug when acting against rheumatoid arthritis [29], we observed a marked inhibition of the activity as expected. However, this inhibition was achieved at much lower concentrations than those needed to achieve a cytotoxic effect, suggesting that the inhibition of TrxR is not sufficient to kill HGSOC cells. Similarly, TrxR activity was inhibited by auranofin at non-cytotoxic doses in Calu-6 lung cancer cells [47]. However, other studies have found that the auranofin inhibition of TrxR in various cancers requires cytotoxic concentrations, including the endometroid ovarian carcinoma cell line A2780 [19,47,64,67,68,69]. Notably, in our studies, the inhibition of TrxR was lower in the PEO4 cells than in the PEO1 cells, and the PEO4 cells also displayed less basal TrxR1 activity. This may explain the greater resistance of the PEO4 cells to auranofin cytotoxicity, as they may rely less on the TrxR antioxidant system for survival when compared to the PEO1 cells. The inhibition of TrxR is a well-documented cause of oxidative stress [70]. When confirming the TrxR inhibition by auranofin in HGSOC, we showed that the drug elevated ROS production in the PEO1 and PEO4 cells and the increase was greater in the PEO1 cells. We also noted that the basal levels of ROS were higher in the platinum-resistant PEO4 cells. This observation is in agreement with evidence that a high basal expression of ROS associates with an increased resistance to platinum [71]. These elevated levels of ROS are tolerated via the expression of antioxidant genes resulting from the interaction of mutated p53 with the ROS-sensitive transcription factor nuclear factor erythroid 2-related factor 2 (NRF2) [72,73]. This indicates that p53 mutations found in HGSOC can lead to increased ROS levels with a compensatory elevation of antioxidant activity for protection [71]. 

Once we confirmed that ROS production in HGSOC was induced by auranofin, we then explored whether ROS plays a role in the cytotoxic effects elicited by the gold complex. We used N-acetyl cysteine (NAC), an agent that reduces ROS indirectly via the upregulation of NRF2 and increases the synthesis of the antioxidant glutathione (GSH) by providing cysteines [45]. NAC can also scavenge ROS molecules directly when it is metabolized into sulfane sulfur species thus exhibiting a cytoprotective role [74]. NAC reversed the lethality and the detrimental morphological effects induced by auranofin in the PEO1 and PEO4 cells, demonstrating that these cytotoxic effects are primarily dependent on ROS-induced damage. 

In most of the parameters of cytotoxicity that we assessed, the PEO4 cells were less sensitive to auranofin than the PEO1 cells. Interestingly, however, when analyzing the mitochondrial membrane potential in response to auranofin, the PEO4 cells show a higher fraction of cells with dissipation of the mitochondrial potential compared to the PEO1 cells. The mitochondrial membrane potential plays a critical role initiating death as part of the intrinsic apoptotic pathway [75]. Studies performed in platinum-resistant NSCLC cells and OV2008 C13* cells showed an increased mitochondrial mass in comparison to the sensitive cell lines [76,77]. In contrast, other studies found that cisplatin-sensitive ovarian cancer cells contain increased mitochondrial content and mitochondrial ROS in comparison to those less sensitive to cisplatin [78,79]. Thus, controversy exists concerning the role of mitochondrial content on platinum sensitivity. In our case, the platinum-resistant PEO4 cells may have higher mitochondrial function, reflected by their increased sensitivity to the depolarization of the mitochondrial membrane by auranofin in comparison to the platinum-sensitive PEO1 cells. Notably, the depolarization of the mitochondrial membrane by auranofin in the PEO1 and in PEO4 cells was dependent on the production of ROS, as shown by others [80]. 

In addition to the disruption of the mitochondrial membrane potential by auranofin, we also found a differential increase in the activation of the executor caspases-3/7 and the cleavage of PARP in the PEO1 and PEO4 cells, with the effect being greater in the platinum-sensitive PEO1 cells. The activation of caspase-3/7 by auranofin has also been reported in mutant p53 NSCLC cells [30], in p53-null SKOV3 ovarian cancer cells [23], in human gastric cancer cells [55], and in chronic lymphocytic leukemia (CLL) [81]. The cell death induced by auranofin in HGSOC was not dependent on the activation of executer caspases as the decrease in viability was not prevented by the presence of a pan-caspase inhibitor (Figure S1). Additionally, the increased caspase-3/7 activity and PARP cleavage induced by auranofin was dependent on ROS production as it was blocked by NAC. Similarly, the cleavage of caspase-3 and PARP by auranofin is ROS-dependent in A549 human lung cancer cells [47], gastric cancer cells [55], and in CLL [81]. 

Apoptosis commonly occurs as a secondary response to sufficient DNA damage to prevent the survival of cells with genomic instability [82]. Since we found that auranofin elicited caspase-3/7-associated apoptosis in HGSOC, we explored the occurrence of DNA damage and detected an increase, which was more significant in the PEO1 cells than in the PEO4 cells, following short-term exposure to auranofin. The higher sensitivity of the PEO1 cells was likely due to their defective homologous recombination DNA repair machinery and germline mutation in *BRCA2* [28]. Accordingly, the decreased sensitivity of the PEO4 cells to DNA damage may be primarily due to their functional DNA repair machinery resulting from the reversion of the *BRCA2* mutation [83], which confers platinum resistance by restoring genome stability allowing the cancer cells to proliferate [84]. The DNA damage induced by auranofin was ROS-dependent, as it was prevented by the antioxidant NAC. A similar finding was observed in acute lymphoblastic leukemia (ALL), in which auranofin induced DNA damage by increasing the ROS levels [57]. 

Aside from the cytotoxic effects already mentioned, auranofin has been proposed to be a proteasomal deubiquitinase inhibitor [29,51,85]. The proteasome degradation cycle is heavily used by cancer cells to regulate protein homeostasis, making this pathway an attractive therapeutic target in cancer [86]. In this cycle, proteins that are meant for degradation are tagged with ubiquitin [87]. Interestingly, we detected an accumulation of polyubiquitinated proteins in the PEO1 and PEO4 cells following treatment with auranofin and demonstrated that such ubiquitination is blocked by NAC, indicating that auranofin-induced polyubiquitination is dependent on ROS production (Figure S2). It is unknown from our results, however, whether the polyubiquitination is a consequence of proteasomal inhibition by auranofin, or, as reported by others, because of the inhibition of a deubiquitinase enzyme [39].

Since we demonstrated that the PEO4 cells were less sensitive to auranofin than the PEO1 cells, we conclude that auranofin as a monotherapy may not be sufficient for treating recurrent stages of HGSOC associated with platinum resistance. Auranofin has been shown to elicit anti-cancer effects in various combination treatments, which have been studied in human lung cancer, malignant B cells, breast cancer, gastric cancer, non-HGSOC ovarian cancer, brain tumor cells, and NSCLC (revisited in [33]). However, few studies have combined auranofin with another agent to target HGSOC cells. To develop a proficient consolidation therapy against HGSOC, we combined auranofin with another pro-oxidant: L-buthionine sulfoximine (L-BSO). L-BSO is an inhibitor of the rate-limiting enzyme, *γ*-glutamcysteine synthetase, which is involved in the synthesis of glutathione (GSH) [88]. At moderate levels, GSH plays protective roles within the cell, including the removal of ROS, regulation of the cell cycle, and regulation of apoptosis and necrosis [89]. Elevated levels of GSH have been detected in various cancers, including breast, ovarian, and lung [90] in association with tumor progression and drug resistance, including resistance to cisplatin [91]. Thus, GSH is an attractive target in platinum-resistant cancer cells, so we combined auranofin with L-BSO to block GSH and simultaneously target both antioxidant systems, TrxR and GSH, in HGSOC. This drug combination is also of interest in mesothelioma, lung cancer, rhabdomyosarcoma, and pancreatic cancer [34,47,68,92]. In HGSOC, we documented an increase in GSH in both the PEO1 and PEO4 cells after treatment with auranofin, suggesting that the cells may compensate for the oxidative environment caused by the block of TrxR. However, the addition of L-BSO reduced the elevation of GSH, an effect that was associated with further lethality compared to auranofin alone. The addition of L-BSO also led to higher levels of ROS than those caused by auranofin alone. Of particular interest, L-BSO alone was not able to increase ROS in PEO1 cells, but it did in PEO4 cells. This difference could be related to the higher basal expression of the antioxidant TrxR in PEO1 cells or because PEO4 cells handles higher basal levels of ROS. In summary, compared with monotherapy, the combination of auranofin and L-BSO increased the levels of ROS beyond those triggered by auranofin alone, regardless of the cellular sensitivity to platinum. This suggests that combining these drugs to inactivate both major antioxidant systems may be valuable as a chronic treatment to overcome platinum resistance in HGSOC. 

## 5. Conclusions

**Figure 10:**
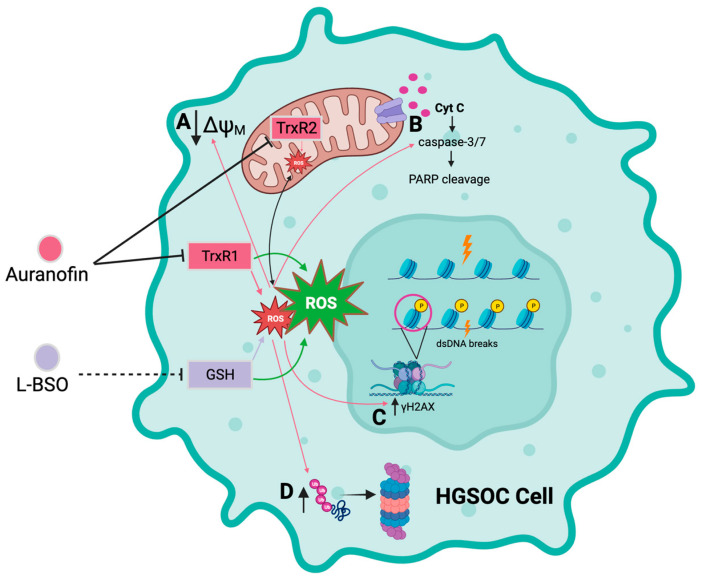
Diagrammatic model depicting the cytotoxicity of auranofin alone or in combination with L-BSO in HGSOC cells. Auranofin (AF) inhibits the activity of the antioxidant enzymes thioredoxin reductase 1 (TrxR1) and 2 (TrxR2), inducing an increase in reactive oxygen species (ROS). In turn, increased ROS induced by AF causes; **A** decreased membrane potential in the mitochondrial membrane, **B** increased activation of the executor caspase-3/7 and cleavage of poly-ADP ribose polymerase (PARP), **C** double-stranded DNA (dsDNA) breaks and phosphorylation of the serine 139 residue of the histone H2AX (γH2AX), and **D** accumulation of polyubiquitinated proteins. L-buthionine sulfoximine (L-BSO) indirectly inhibits the production of the antioxidative protein, glutathione (GSH), resulting in an increase in ROS. The combination of AF and L-BSO results in a further increase in the production of ROS compared to the amount of ROS produced by each drug separately. Arrows in pink correspond to the effects caused by ROS as induced by AF alone. Arrows in green indicate a greater induction of ROS by the combination of AF and L-BSO. Black arrows in **A**,**C**,**D** indicates either increase or decrease in magnitude. Created with BioRender.com.

We report that the gold complex auranofin is efficient in impairing the functionality of HGSOC cells that are clinically sensitive or resistant to cisplatin (Figure 10). We further show that the drug was more efficient in killing the platinum-sensitive cells. The mechanism of cell death induced by auranofin involves the inhibition of TrxR, depolarization of the mitochondrial membrane, production of ROS, and caspase-associated apoptosis linked to DNA damage. This toxicity can be blocked by the antioxidant NAC, indicating the relevance of ROS in the toxicity of auranofin. Furthermore, we provide evidence that in compensation for the pro-oxidant effect of auranofin, there is upregulation of GSH, which if blocked with L-BSO, diminishes the antioxidant systems and potentiates the toxicity of auranofin in both platinum-sensitive and platinum-resistant HGSOC cells. We anticipate that auranofin can be repurposed to chronically treat HGSOC after initial cytotoxic chemotherapy as a maintenance therapy alone or in combination with the antioxidant L-BSO. 

## Data Availability

The detailed data generated in the manuscript are openly available to the scientific community upon request. The content of this manuscript was deposited in the bioRxiv server for biology; bioRxiv preprint posted 17 September 2023: http://doi.org/10.1101/2023.09.13.557629.

## Supplementary Materials

The following supporting information can be downloaded at: https://www.mdpi.com/article/10.3390/cancers15215136/s1, Figure S1: Induction of apoptosis by auranofin is not prevented by caspase inhibition; Figure S2: Auranofin induces accumulation of polyubiquitinated proteins, an effect prevented by NAC; Figure S3: Original membranes containing detailed information of the uncropped immunoblot images.

## Abbreviations

∆*ψ*M	Transmembrane potential
7-AAD	7-Aminoactinomycin D
AF	Auranofin
ALL	Acute lymphoblastic leukemia
ATP	Adenosine triphosphate
BCA	Bicinchoninic acid
BRCA2	Breast cancer type 2 susceptibility protein
BZ	Bortezomib
CI	Combination index
CLL	Chronic lymphocytic leukemia
CO_2_	Carbon dioxide
DEVD	DNA-binding peptide; substrate for caspase-3
DHE	Dihydroethidium
DNA	Deoxyribonucleic acid
DTNB	5,5′-dithio-bis (2-nitrobenzoic acid)
DTT	Dithiothreitol
FBS	Fetal bovine serum
FDA	US Food and Drug Administration
GLOBOCAN	Global Cancer Observatory
GSH	Glutathione
H2AX	Histone variant H2AX
HCl	Hydrochloric acid
HEPES	4-(2-hydroxyethyl)-1-piperazine ethane sulfonic acid
HGSOC	High-grade serous ovarian cancer
HIV	Human immunodeficiency virus
HRP	Horseradish peroxidase
IC50	Half-maximal inhibitory concentration
IgG	Immunoglobulin G
L-BSO	L-buthionine sulfoximine
MTT	3-(4,5-dimethylliazol-2-yl)-2,5-diphenyltetrazolium bromide
NAC	N-acetyl-L-cysteine
NADPH	Nicotinamide adenine dinucleotide phosphate
NRF2	Nuclear factor erythroid 2-related factor 2
NSLC	Non-small cell lung cancer
OD	Optical density
PARP	Poly-adenosine diphosphate (ADP) ribose polymerase
PBS	Phosphate-buffered saline
PE	Phycoerythrin
P3Cy5	Phycoerythrin-Cyanine^®^5
PFA	Paraformaldehyde
PMSF	Phenylmethylsulfonyl fluoride
PS	Phosphatidylserine
ROS	Reactive oxygen species
SDS	Sodium dodecyl sulfate
TBS-T	Tris-buffered saline 0.1% Tween 20
TNB	5′-thio-2-nitrobenzoic acid
TrxR	Thioredoxin reductase
z-DEVD-fmk	Caspase-3 inhibitor
