# Supplementary material for: Auranofin Induces Lethality Driven by Reactive Oxygen Species in High-Grade Serous Ovarian Cancer Cells
Source: Cancers (Basel). 2023 Oct 25;15(21):5136. doi: 10.3390/cancers15215136 (PMC10650616; doi:10.3390/cancers15215136)
Supplement: Supplementary file 1 [file cancers-15-05136-s001.zip › cancers-2670603-supplementary.pdf]

Supplementary Figure S1: Induction of apoptosis by auranofin is not prevented by caspase inhibition

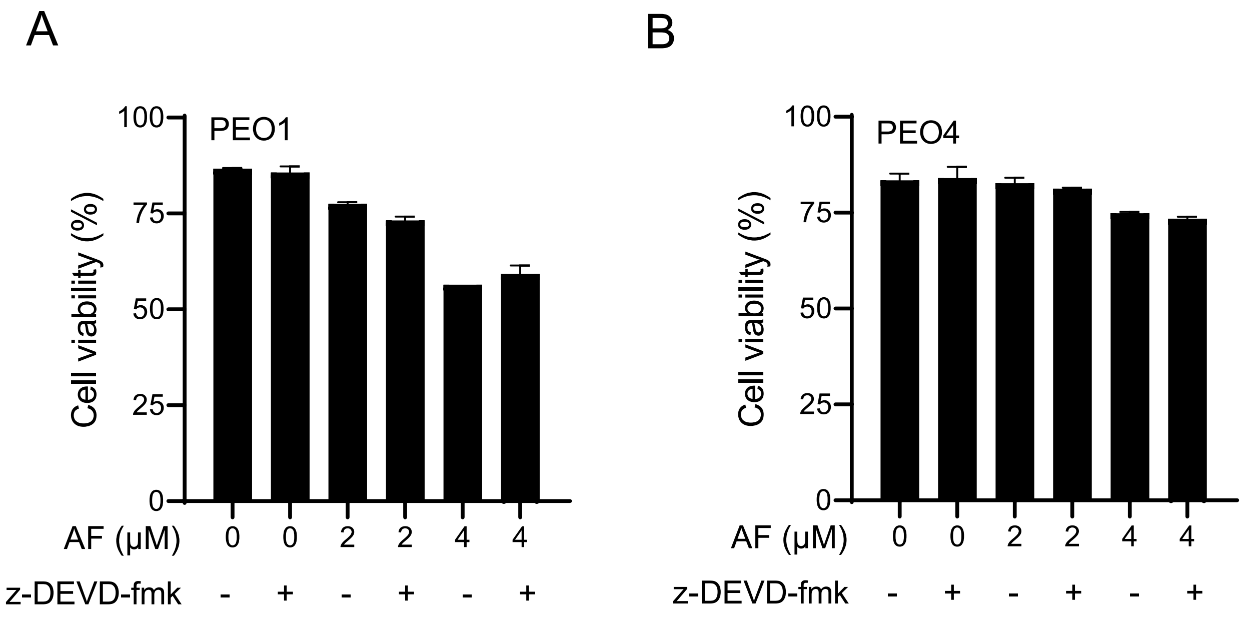

Supplementary Figure S2: Auranofin induces accumulation of polyubiquitinated proteins, an effect prevented by NAC

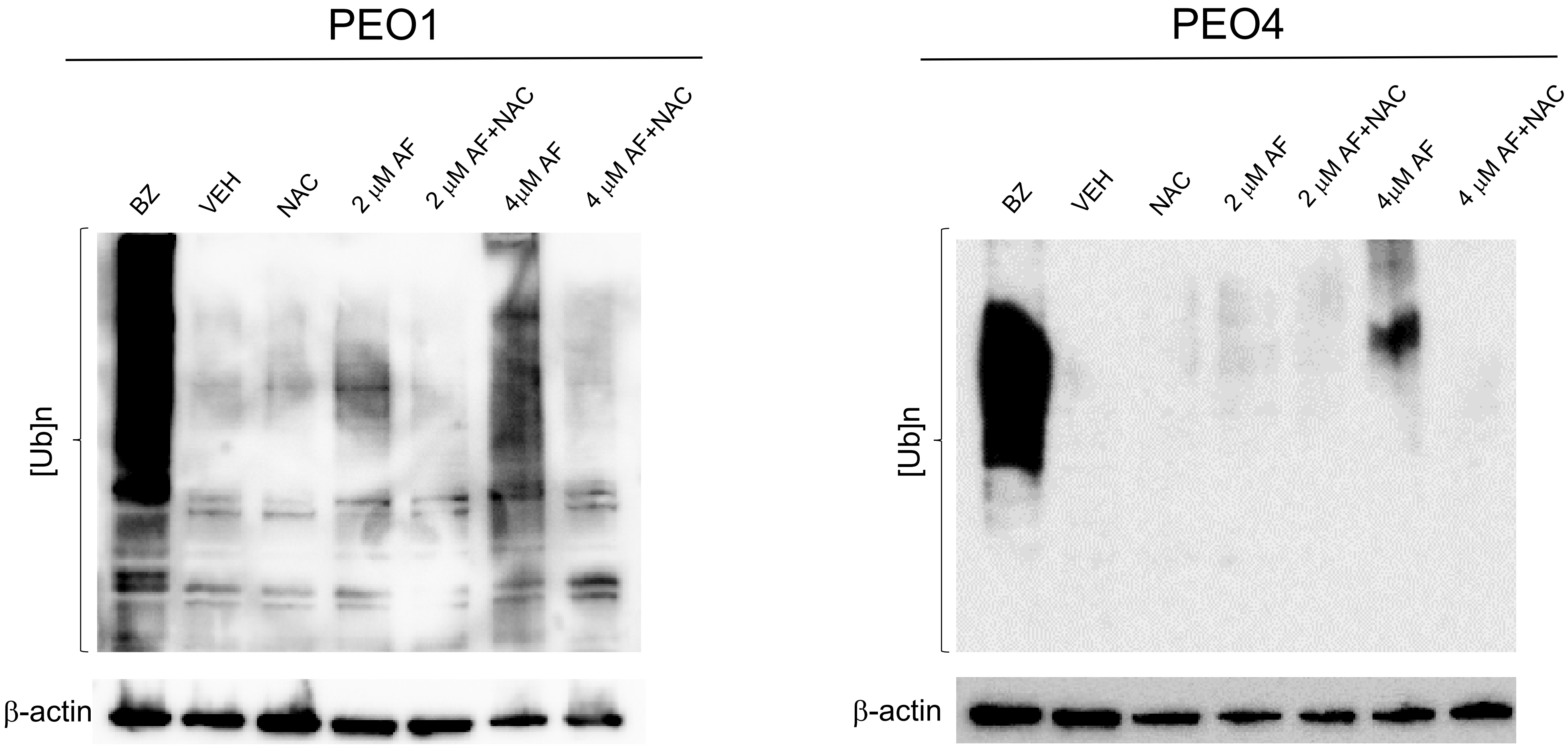

Supplementary Figure S3: Original membranes containing detailed information of the uncropped immunoblot images

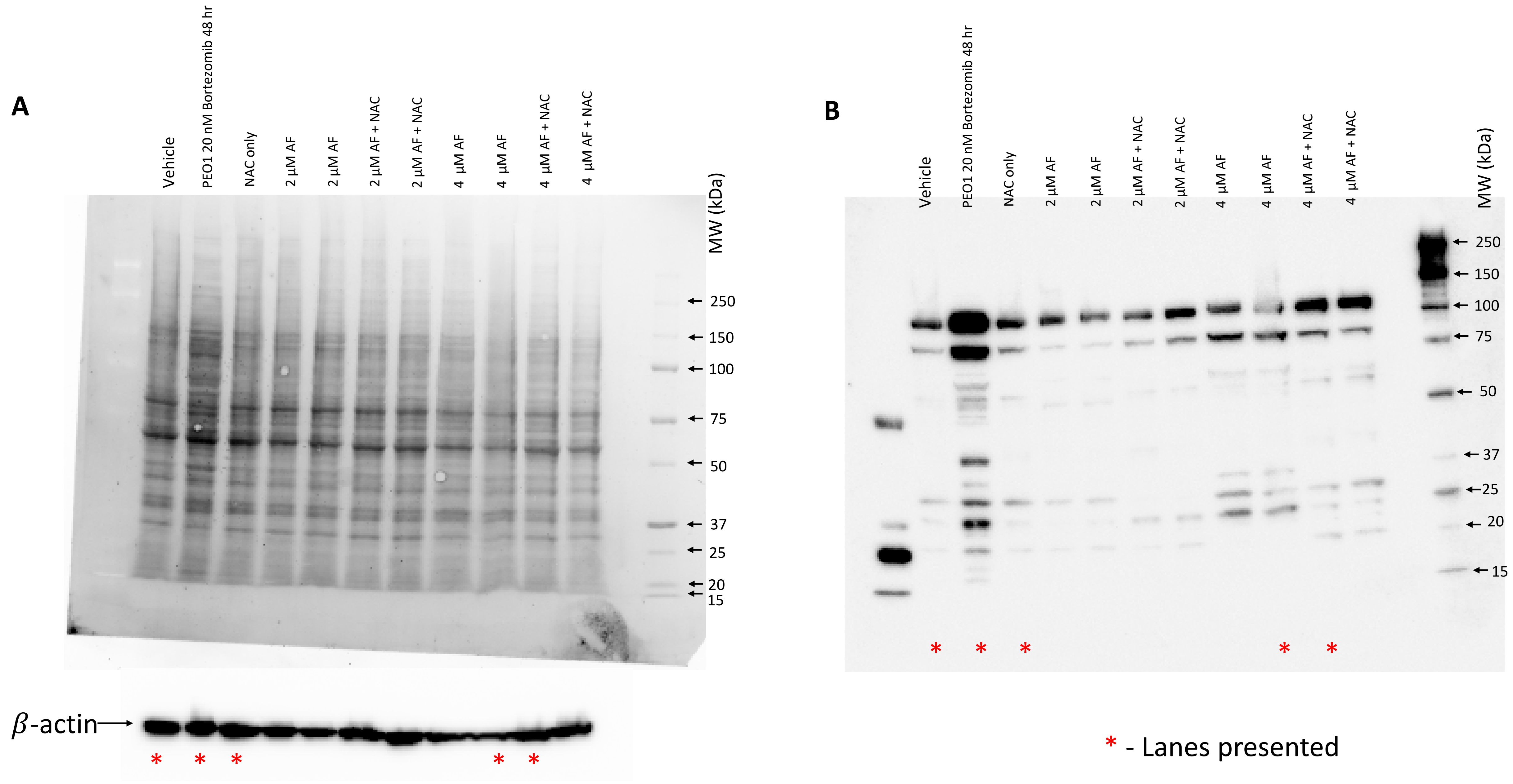

Blot S1. PEO1 cells were treated with the indicated concentrations of AF with or without the presence of 5 mM NAC for 24 hr. PEO1 cells treated with 20 nM bortezomib (Bz) for 48 hr were used as a positive control. Extracted proteins from the samples were run for 35 min at 200V on TGX stain free fast cast acrylamide gel (10 %). After the run, the gel was activated by UV light, and the proteins on the gel were transferred for 7 min using TransBlot Turbo. After the transfer, total protein load was visualized in the unstained membrane using BioRad ChemiDoc imager (A). After 1 h of blocking with 5% non-fat dry milk, the blot was incubated with anti-PARP antibody overnight at 4°C. (B). The blot was then incubated for β-actin (A). Data from this blot was presented in Figure 7B.

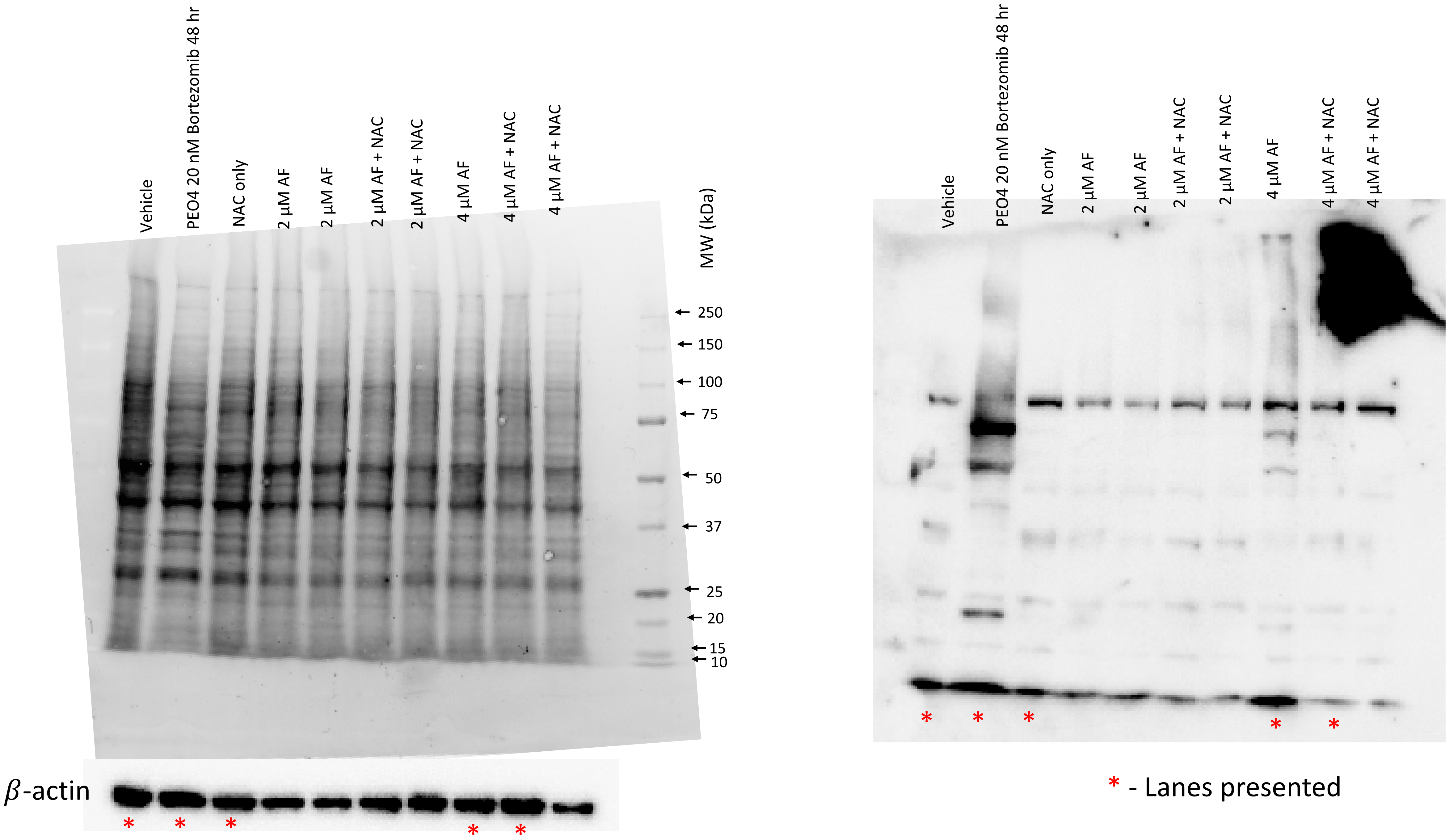

Blot S2. PEO4 cells were treated with the indicated concentrations of AF with or without the presence of 5 mM NAC for 24 hr. PEO4 cells treated with 20 nM bortezomib (Bz) for 48 hr were used as a positive control. Extracted proteins from the samples were run for 35 min at 200V on TGX stain free fast cast acrylamide gel (10 %). After the run, the gel was activated by UV light, and the proteins on the gel were transferred for 7 min using TransBlot Turbo. After the transfer, total protein load was visualized in the unstained membrane using BioRad ChemiDoc imager (A). After 1 h of blocking with 5% non-fat dry milk, the blot was incubated with anti-PARP antibody overnight at 4℃. (B). The blot was then incubated for β-actin (A). Data from this blot was presented in Figure 7D.

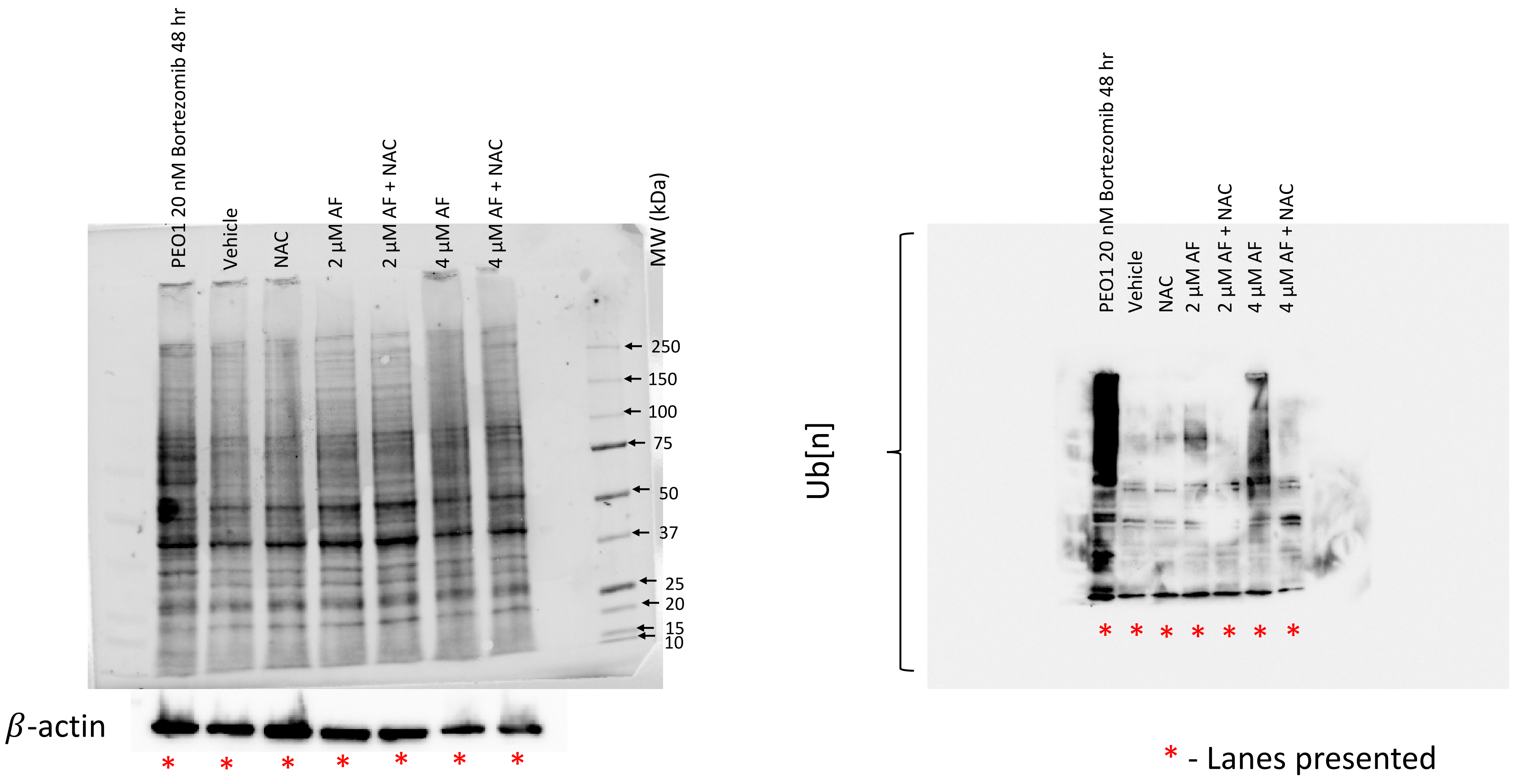

Blot S3. PEO1 cells were treated with the indicated concentrations of AF with or without the presence of 5 mM NAC for 24 hr. PEO1 cells treated with 20 nM bortezomib (Bz) for 48 hr were used as a positive control. Extracted proteins from the samples were run for 35 min at 200V on TGX stain free fast cast acrylamide gel (10 %). After the run, the gel was activated by UV light, and the proteins on the gel were transferred for 7 min using TransBlot Turbo. After the transfer, total protein load was visualized in the unstained membrane using BioRad ChemiDoc imager (A). After 1 h of blocking with 5% non-fat dry milk, the blot was incubated with anti-ubiquitin antibody overnight at 4°C. (B). The blot was then incubated for β-actin (A). Data from this blot was presented in Supplementary Figure S2.

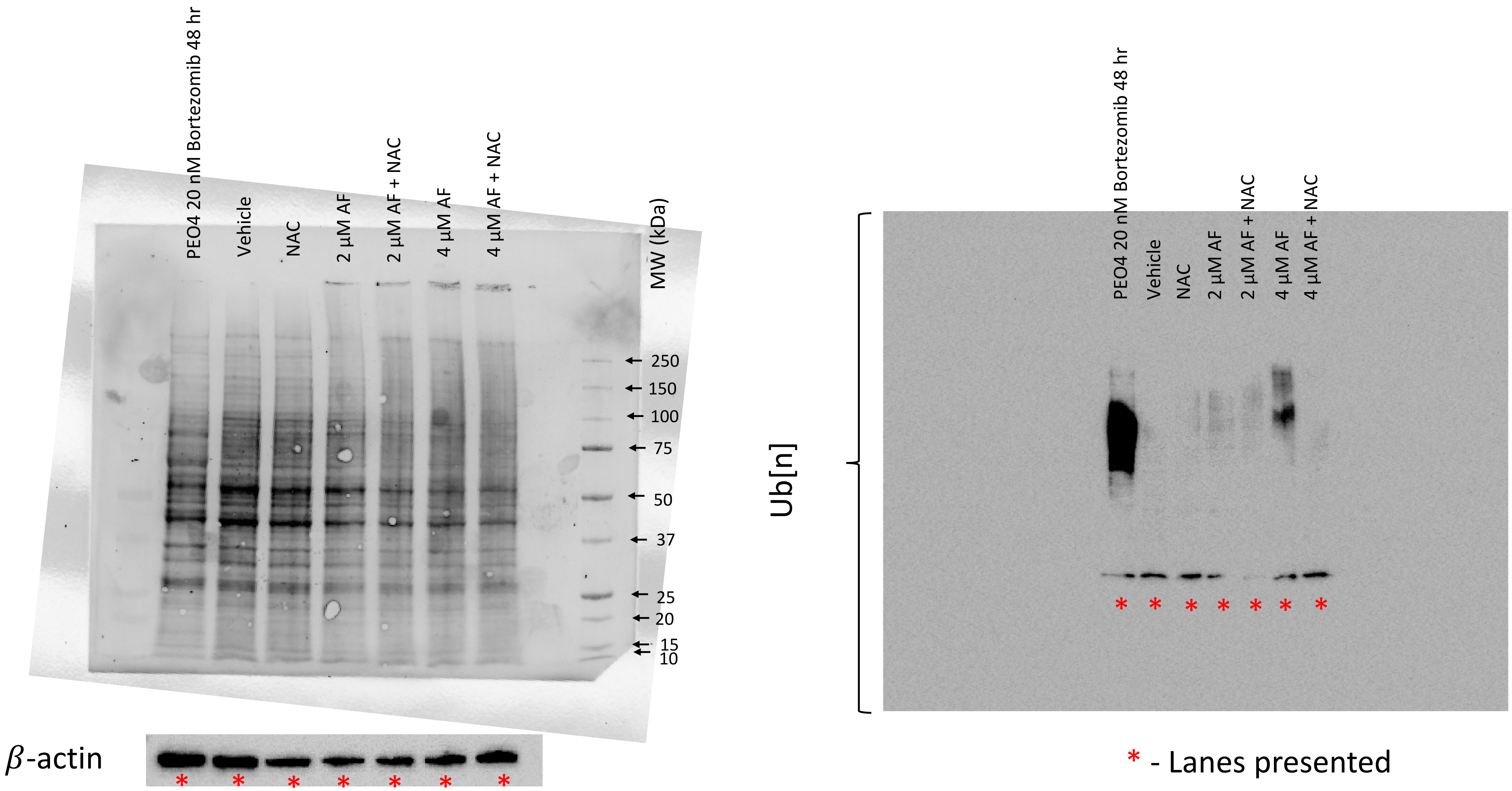

Blot S4. PEO4 cells were treated with the indicated concentrations of AF with or without the presence of 5 mM NAC for 24 hr. PEO4 cells treated with 20 nM bortezomib (Bz) for 48 hr were used as a positive control. Extracted proteins from the samples were run for 35 min at 200V on TGX stain free fast cast acrylamide gel (10%). After the run, the gel was activated by UV light, and the proteins on the gel were transferred for 7 min using TransBlot Turbo. After the transfer, total protein load was visualized in the unstained membrane using BioRad ChemiDoc imager (A). After 1 h of blocking with 5% non-fat dry milk, the blot was incubated with anti-ubiquitin antibody overnight at 4°C. (B). The blot was then incubated for β-actin (A). Data from this blot was presented in Supplementary Figure S2.
